# The rice PALE1 homolog is involved in the biosynthesis of vitamin B1

**DOI:** 10.1111/pbi.13465

**Published:** 2020-09-03

**Authors:** Ping‐Han Hsieh, Yi‐Hsin Chung, Kim‐Teng Lee, Shi‐Yun Wang, Chung‐An Lu, Ming‐Hsiun Hsieh

**Affiliations:** ^1^ Institute of Plant and Microbial Biology Academia Sinica Taipei Taiwan; ^2^ Department of Life Sciences National Central University Taoyuan Taiwan

**Keywords:** vitamin B1, rice, pale green, thiamin, thiamin monophosphate, thiamin monophosphate phosphatase

Vitamin B1 is an essential cofactor for central metabolism in all organisms. In humans, vitamin B1 deficiency is often associated with cardiovascular diseases and neurological disorders. Polished white rice (*Oryza sativa*) is a poor source of vitamin B1. Biofortification of rice with vitamin B1 has the potential to improve human health and nutrition, but the implementation of this strategy requires detailed knowledge about the biosynthesis of vitamin B1, which is poorly studied in rice.

Most, if not all, genes involved in vitamin B1 biosynthesis have been uncovered in the reference plant *Arabidopsis thaliana* (Strobbe and Van Der Straeten, 2018). Vitamin B1 is composed of thiazole and pyrimidine moieties. The thiazole moiety, hydroxyethyl thiazole phosphate (HET‐P), is synthesized from glycine, nicotinamide adenine dinucleotide (NAD) and a sulphide group from HET‐P synthase (THI1) that catalyses the reaction (Chatterjee *et al*., 2011; Fitzpatrick and Thore, 2014). The pyrimidine moiety, hydroxymethylpyrimidine phosphate (HMP‐P), is derived from a complex rearrangement of aminoimidazole ribonucleotide catalysed by HMP‐P synthase (THIC) (Raschke *et al*., 2007). HMP‐P is phosphorylated to HMP‐PP by HMP‐P kinase/thiamin monophosphate (TMP) pyrophosphorylase (TH1), which also catalyses the condensation of HMP‐PP and HET‐P to TMP (Goyer, 2017). TMP synthesized in the chloroplast is dephosphorylated to thiamin, which is then converted to the bioactive thiamin diphosphate (TDP) by thiamin pyrophosphokinase (TPK) in the cytosol (Ajjawi *et al*., 2007).

Recently, a TMP phosphatase encoded by the *PALE1/TH2* gene has been shown to be involved in the dephosphorylation of TMP to thiamin in Arabidopsis (Hsieh *et al*., 2017; Mimura *et al*., 2016). While Mimura *et al*. (2016) proposed that the TMP phosphatase is mainly localized in the cytosol, the data derived from *35S:PALE1‐GFP* complemented *pale1* mutants indicate that Arabidopsis PALE1 is localized to the mitochondrion (Hsieh *et al*., 2017). The Arabidopsis *pale1/th2* mutants are not lethal, indicating that additional TMP phosphatases or nonspecific phosphatases capable of dephosphorylating TMP to thiamin are present in the mutants. Nonetheless, the localization of Arabidopsis TMP phosphatase in the mitochondrion suggests that the conversions among TMP, thiamin and TDP are more complicated than originally thought.

In contrast with Arabidopsis, very few genes involved in vitamin B1 biosynthesis have been characterized in rice. The *OsDR8* gene encodes a THI1 homolog that has dual function in disease resistance and thiamin accumulation (Wang *et al*., 2006). The *ROX1* gene, encoding a positive regulator of XA21 in innate immune response, corresponds to *OsTPK1* (Lee *et al*., 2011). Here, we provide experimental evidence to show that *OsPALE1* (*Os08g0566000*) is involved in vitamin B1 biosynthesis in rice.

To study the function of *OsPALE1*, we used CRISPR/Cas9 genome editing to create knockout mutants. The single‐guide RNA (5′‐CGAGGGAGGCCGCCTTCGCC‐3′) was cloned into a CRISPR/Cas9 vector and transformed into rice. We successfully obtained 10 transgenic plants showing similar phenotypes. Initially, the mutant leaves were pale green, but they gradually turned yellowish brown and eventually died. A representative *Ospale1* mutant plant is shown in Figure 1a.

**Figure 1:**
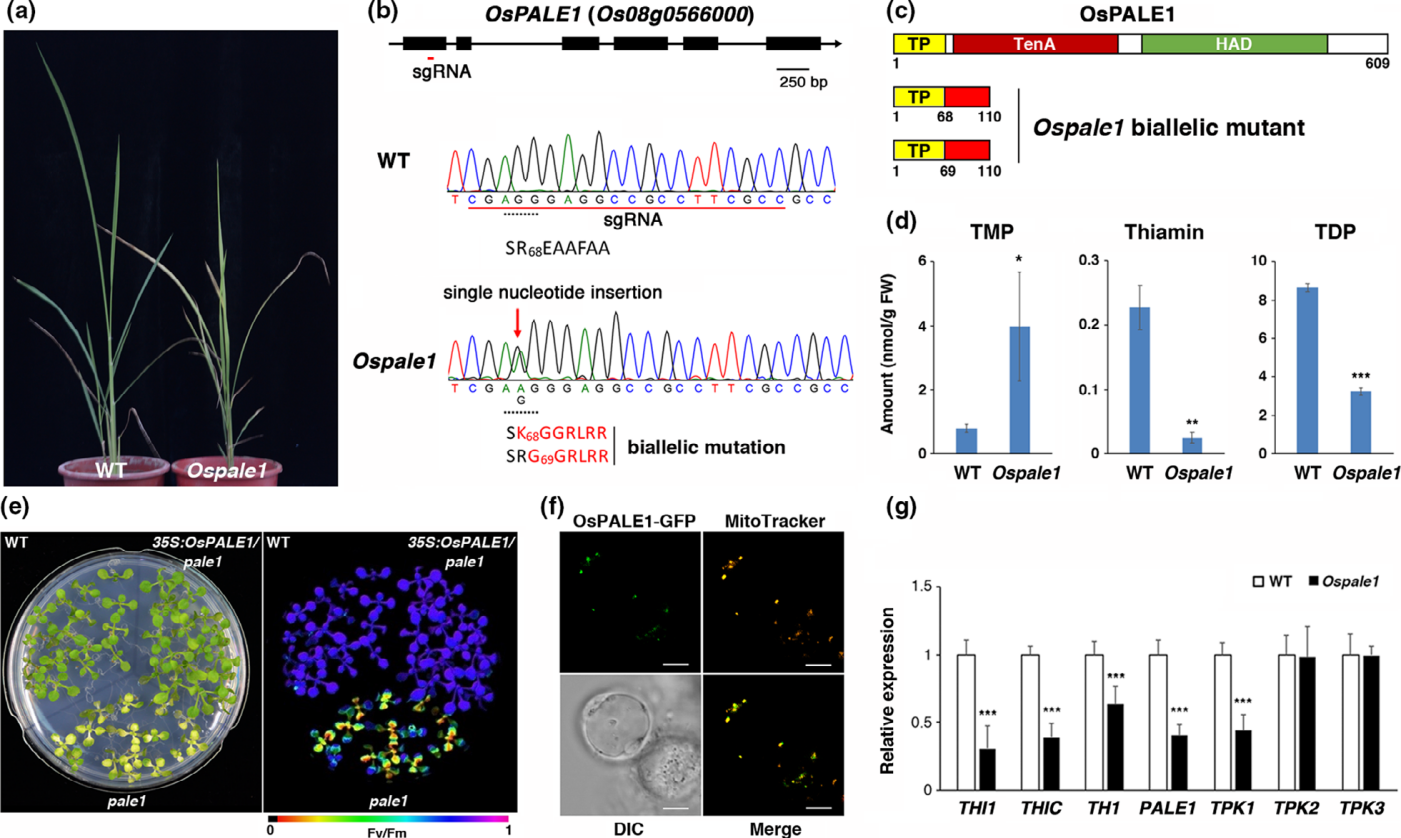
CRISPR/Cas9‐induced mutation in the *OsPALE1* gene. (a) Phenotypes of a representative *Ospale1* mutant compared with the wild type (WT) at a similar developmental stage. (b) Schematic diagram of the *OsPALE1* gene and DNA sequences around the editing site. The encoded peptides are shown at the bottom of the DNA sequences. Red underline indicates the position of the sgRNA. Dashed underline indicates the 68th codon of *OsPALE1*. The 'A' and 'G' insertions in *Ospale1* are indicated by a red arrow. Different amino acids encoded by the mutant alleles are shown in red. (c) Schematic diagrams of the OsPALE1 protein. The biallelic mutation in *Ospale1* results in a frame shift (amino acids 68–110 or 69–110 shown in red) and a premature stop codon. TP, targeting peptide. (d) Levels of thiamin monophosphate (TMP), thiamin and thiamin diphosphate (TDP) in the WT and *Ospale1* mutant leaves. (e) Complementation of Arabidopsis *pale1* by *35S:OsPALE1*. Light (left) and chlorophyll fluorescence (right) images of 12‐day‐old seedlings grown on a tissue culture plate. (f) Localization of OsPALE1‐GFP in mitochondria. The OsPALE1‐GFP signals co‐localized with MitoTracker Orange in the rice protoplasts. The excitation/emission wavelengths (nm) for GFP and MitoTracker Orange are 488/492–540 and 561/571–609, respectively. DIC, differential interference contrast. Scale bars are 10 μm. (g) Quantitative RT‐PCR analysis of vitamin B1 biosynthesis genes in leaves from WT and *Ospale1* plants. Data in (d) and (g) are means ± SE of three biological repeats (Student’s *t‐*test, *P < 0.05, **P < 0.01, ***P < 0.005). [Colour figure can be viewed at wileyonlinelibrary.com]

DNA sequence analyses revealed that all 10 transgenic plants have the same biallelic mutation: an A or G single‐nucleotide insertion at the same site (Figure 1b). Thus, these plants may be derived from the same *Ospale1* line during callus regeneration. OsPALE1 has a targeting peptide followed by the TenA and HAD domains (Figure 1c). The single‐nucleotide 'A' insertion results in a frame shift starting from the 68th amino acid residue and creates a premature stop codon (Figures 1b and c). The 'G' insertion does not change the 68th AGG codon, and the frame shift occurs from the 69th to the 110th amino acids (Figures 1b and c). We also sequenced the reverse transcription PCR products from the *Ospale1* mutant and confirmed that both 'A' and 'G' insertions are present in the *Ospale1* mutant transcripts.

We measured the amounts of TMP, thiamin and TDP in the wild‐type (WT) and *Ospale1* leaves with a modified method described previously (Hsieh *et al*., 2017). The level of TMP in the *Ospale1* mutant was about fivefold of the WT (Figure 1d). By contrast, the levels of thiamin and TDP in the *Ospale1* mutant were 11% and 37% of the WT, respectively (Figure 1d). In addition to the characterization of the *Ospale1* mutant, we also confirmed that *35S:OsPALE1* was able to complement the Arabidopsis *pale1* mutant (Figure 1e). The phenotypes of the *pale1* mutant (Hsieh *et al*., 2017) were fully restored in the complementation lines. Taken together, these results support the notion that OsPALE1 is involved in the conversion of TMP to thiamin in vitamin B1 biosynthesis.

The *35S:OsPALE1‐GFP* construct was transformed into rice protoplasts for subcellular localization assays. The OsPALE1‐GFP is localized to the mitochondrion (Figure 1f), which is consistent with Arabidopsis PALE1‐GFP (Hsieh *et al*., 2017). We could not exclude the possibility that OsPALE1 is dually localized in the cytosol and mitochondria. The usage of the 35S promoter may dispropotionately enrich the fusion protein in the mitochondrion. Nevertheless, an *Ospale1* mutant complemented by the *OsPALE1p:OsPALE1‐GFP* construct driven by a native promoter may provide a better answer for the subcellular localization of OsPALE1 *in planta*.

We used quantitative RT‐PCR analysis to compare the expression levels of vitamin B1 biosynthesis genes in WT and *Ospale1* leaves. While the expression of *THI1* (*Os07g0529600*), *THIC* (*Os03g0679700*), *TH1* (*Os12g0192500*) and *TPK1* (*Os01g0931400*) was down‐regulated, the expression of *TPK2* (*Os01g0356500*) and *TPK3* (*Os05g0367400*) was not affected in the mutant (Figure 1g). The abundance of the *Ospale1* mutant transcripts in the mutant was lower than that of the *OsPALE1* transcript in the WT (Figure 1g), which is consistent with the notion that transcripts with a premature termination codon will be selectively degraded by the non‐sense‐mediated mRNA decay pathway.

The pale‐green to yellowish‐brown phenotype of the *Ospale1* knockout mutant is reminiscent of the Arabidopsis thiamin‐deficient mutant *pale1* (Hsieh *et al*., 2017). Still, there are at least two distinct aspects of OsPALE1. First, *OsPALE1* is an essential gene in rice. The TDP levels in the *Ospale1* leaves are approximately 40% of the WT (Figure 1d), and the mutant is lethal. Similarly, the TDP levels in the Arabidopsis *pale1* mutant seedlings are also about 40% of the WT, but the mutant is not lethal (Mimura et al., 2016; Hsieh *et al*., 2017). These results suggest that rice may have a stricter demand for TDP to complete its life cycle. Second, Arabidopsis and rice may have distinct mechanisms to regulate the expression of vitamin B1 biosynthesis genes. The expression of vitamin B1 biosynthesis genes was up‐regulated in the Arabidopsis *pale1* mutant (Hsieh *et al*., 2017). By contrast, a blockage in the conversion of TMP to thiamin results in down‐regulation of vitamin B1 biosynthesis genes in rice (Figure 1g). Further studies on vitamin B1 biosynthesis genes and their regulation in rice may have practical application in agriculture and nutrition in the future.

## Conflicts of interest

The authors declare no conflicts of interest.

## **Author** **contributions**

M.H. Hsieh conceived and designed the experiments and wrote the paper. P.H. Hsieh, Y.H. Chung and K.T. Lee performed the experiments and analysed the data. S.Y. Wang and C.A. Lu generated the transgenic plants.
